# Safety aspects in surgical treatment of pathological fractures of the proximal femur – modular endoprosthetic replacement vs. intramedullary nailing

**DOI:** 10.1186/1754-9493-7-37

**Published:** 2013-12-07

**Authors:** Johannes KM Fakler, Franziska Hase, Jörg Böhme, Christoph Josten

**Affiliations:** 1 Department of Orthopaedic Trauma, Reconstruction and Plastic Surgery, University Hospital Leipzig, University of Leipzig, Liebigstr. 20, 04103 Leipzig, Germany

**Keywords:** Pathologic fractures, Femoral metastasis, Endoprosthetic reconstruction, Intramedullary nailing, Survival

## Abstract

**Background:**

Pathologic fractures of the femoral intertrochanteric and subtrochanteric region require special consideration in terms of biomechanically stable fixation and durability of the implant. In addition, the type of surgery might also influence patient survival. We conducted this retrospective study to evaluate the safety of modular proximal femur replacement compared to intramedullary nailing with patient survival being the primary and complications the secondary endpoint.

**Methods:**

We retrospectively studied the records of 20 consecutive patients with actual pathologic fracture due to bone metastasis in the intertrochanteric and subtrochanteric part of the femur. The pathologic fractures were stabilized with a locked cephalomedullary nail in 12 patients and treated with en-bloc resection and modular proximal femur replacement in eight patients.

**Results:**

In the tumor prosthesis group median patient survival was more than twice as high (4.5 months, IQR 2.3 – 16.5) than in the osteosynthesis group (2.0 months, IQR 0.3 – 20.5), but did not reach significance (p = 0.58). Besides, a significantly better preoperative general health status in patients with endoprosthetic reconstruction puts better survival into perspective. Median implant survivorship did not differ between groups with 2.5 (IQR 1.0 – 7.5) months for endoprothesis and 3.0 (IQR 0.3 – 11.0) months for osteosynthesis (p = 0.93). Complication rates were comparable with 25% in each group.

**Conclusion:**

Patient survival was not influenced by type of surgery or choice of implant. Preoperative general health condition and ambulatory capacity may aid in decision for type of surgery and improve patient safety, respectively.

## Background

The most frequent site of extravertebral osseous metastatic lesions is located in the femur, specifically in the proximal part of it [1-3]. Bone metastases of the femoral head and neck with subsequent fractures usually are treated with conventional arthroplasty [4]. On the contrary, pathologic fractures of the trochanteric region not only necessitate restoration of hip function, but also demand full weight-bearing capacity to the femoral diaphysis. Hence, pathologic fractures of the femoral intertrochanteric and subtrochanteric region require special consideration in terms of biomechanically stable fixation of the implant and restoration of lower limb function [4,5]. Since healing of pathologic fractures can be expected in only about 35% of all pathologic fractures [6], durable reconstitution of load capacity in this biomechanically critical region must be provided by the implant itself in many cases. Apart from technical aspects, variable general health condition and indistinct survival time of patients with secondary osseous tumor lesions [4,5,7-9] impede decision making in terms of optimal surgical procedure and choice of implant. Although previous studies reported on strategies and outcome, optimal treatment is still under debate. Recently it was demonstrated, that patient survival may benefit from resection and modular replacement with a tumor prosthesis compared to intramedullary nailing in pathologic fracture of the proximal femur [5]. We conducted this retrospective study to evaluate the safety of modular proximal femur replacement compared to intramedullary nailing in the treatment of pathologic trochanteric femoral fracture with respect to survival time of patients and implants as well as complications.

## Methods

We retrospectively studied the records of 20 consecutive patients with pathologic fractures due to bone metastasis in the intertrochanteric and subtrochanteric part of the femur at the author’s institution from January 2003 to December 2012. Patients with an impending fracture as well as patients with a solitary metastasis were excluded for better reproducibility. All patients gave written consent for scientific analysis of their data. All bone metastases were confirmed by biopsy. All values are given as median values and the interquartile range IQR (25^th^-75^th^ percentile). Median age of all patients was 69.8 years (IQR 61.8 – 74.0). Nine patients were female and eleven male. The median postoperative follow-up was 3.0 months (IQR 1.0 – 18.3). No patient was lost to follow-up. In order to estimate the general health condition preoperatively the Karnofsky performance status was used [10]. A performance status of 80-100% was regarded as a good general health condition, 50-79% as moderate and 10-49% as poor. Walking ability was subdivided in three groups: ambulatory without any walking aids, ambulatory with walking aids and not ambulatory (wheel-chair or bed-bound). 15 of 20 patients (75%) also had vertebral metastases at the time of pathologic proximal femoral fracture. Hence, the Tokuhashi-Score was calculated to predict patient survival [11].

**Figure 1:**
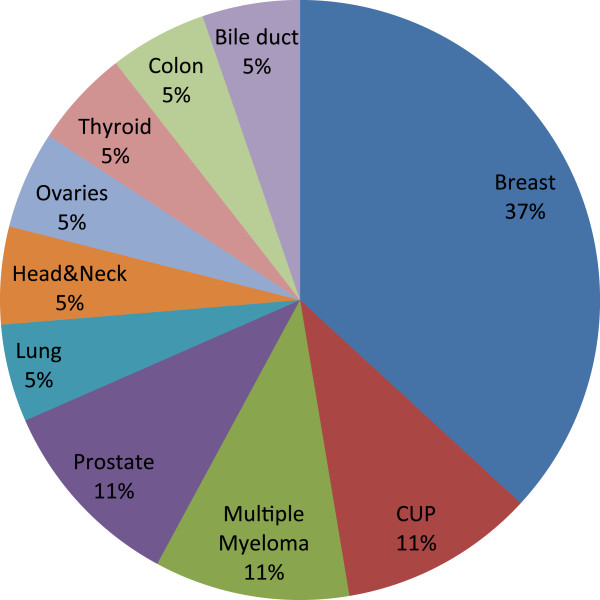
Distribution of cancer types.

The breast was the most common site of the primary tumor (35%), followed by prostate cancer, multiple myeloma and cancer of unknown origin with each 10% (Figure 1).

### Operative treatment

The pathologic fractures were stabilized with a locked cephalomedullary nail in 12 patients in the osteosynthesis group and treated with en-bloc resection and modular proximal femur replacement in eight patients in the tumor prosthesis group. With respect to osteosynthetic stabilization a proximal femoral antirotation nail with a spiral blade (PFNA, Synthes, Oberdorf, Switzerland) was used in two cases and cephalomedullary nail (Sirus, Zimmer, Freiburg, Germany) in the remaining ten patients. In three patients curettage and cementation was performed additionally. Two patients primarily treated with a cephalomedullary nail were converted to a cemented proximal femoral replacement after early failure of the osteosynthesis three and five months after operation (cut out of the cephalic screw and implant breakage). In eight patients the tumor prosthesis was implanted primarily. All patients with resection received a cemented modular proximal femoral replacement (MUTARS, Implantcast, Buxtehude, Germany). Hemiarthroplasty as well as total arthroplasty was performed in four patients. A lateral approach was performed in all proximal femoral replacements.

### Survival analysis

The patient survival was assessed by the time interval from operation until death or last follow-up for patients alive. Implant survivorship was determined as the time period from the operation until death, last follow-up of patients alive or re-operation for any reason at the same site of the operation. Implant durability was defined as the time period from operation until death, last follow-up, or implant exchange due to structural failure as for secondary fracture dislocation, periimplant/periprosthetic fracture, hardware failure or loosening.

### Statistical analysis

Patient survival was defined as primary outcome with implant survival and complications rates being secondary endpoints. Correspondingly, our primary hypothesis was that patient and implant survival is higher for megaendoprosthetic replacement compared to osteosynthesis. Due to the retrospective nature of this study structural equality of groups cannot be assumed, subsequently a power-analysis was not performed. Statistical analysis was performed with the PASW software version 20 (SPSS Inc., Chicago, IL, USA). Comparison and testing for differences in both groups was assessed with the Mann-Whitney-U test. For patient and implant survival the Kaplan-Meier analysis was applied, differences were determined by log-rank analysis. Differences were considered statistically significant when the p value was less than 0.05.

## Results

Survival of all patients at six and twelve months was 45.0% and 35.0%, respectively. At two years three patients were alive (15.0%). At the last follow-up three patients were still alive 22, 35 and 45 months after operation.

**Figure 2:**
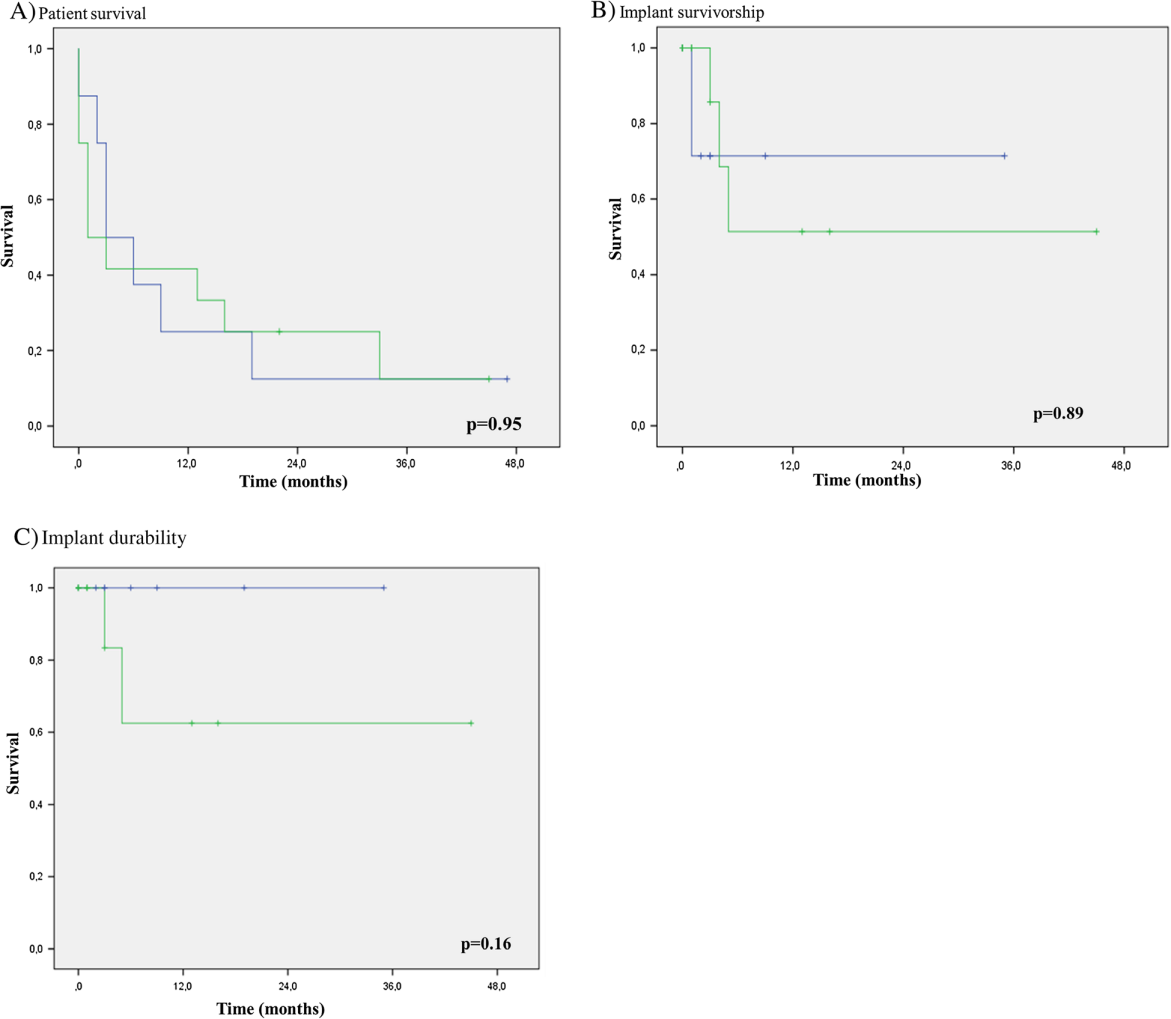
**Survival curves. A)** Patient survival. Kaplan-Meier curves showing survival for patients with proximal femoral replacement (blue) and osteosynthesis (green). **B)** Implant survivorship. Kaplan-Meier curves demonstrating survivorship of proximal femoral replacement (blue) and osteosynthesis with revision surgery for any reason at the site of primary surgery as an endpoint. **C)** Implant durability. Kaplan-Meier curves demonstrating durability of proximal femoral replacement (blue) and osteosynthesis with implant exchange due to structutal implant failure.

The overall median preoperative Karnofsky performance status was 50% (IQR 40.0 – 77.5) and the Takahashi Score 6.5 (IQR 6.0 – 8.0). Median length of operation was 135 minutes (IQR 101 – 179) in the arthroplasty group compared to 81 minutes (IQR 56 – 123) in the osteosynthesis group (Table 1). In the tumor prosthesis group median patient survival was more than twice as high (4.5 months, IQR 2.3 – 16.5) than in the osteosynthesis group (2.0 months, IQR 0.3 – 20.5), but did not reach significance (p = 0.58). Median implant survivorship which takes any complication into account, did not differ between groups with 2.5 (IQR 1.0 – 7.5) months for endoprothesis and 3.0 (IQR 0.3 – 11.0) months for osteosynthesis (p = 0.93). With respect to implant durability, referring only to complications associated with the structural integrity of the implant (i.e. breakage), superiority was seen for the endoprosthesis (median 4.5, IQR 2.3 – 16.5) compared to the intramedullary nail (median 2.0, IQR 0.3 – 11.0 months), but the difference was also not significant (p = 0.31). Kaplan-Meier analysis for patient survival, implant survivorship and durability also failed to demonstrate a significant difference (Figure 2). Median time from first tumor diagnosis until fracture was 24.0 months (IQR 1.0 – 64.3) in the osteosynthesis group and 54.5 months (IQR 4.8 – 87.3) in the tumor prosthesis group (p = 0.43). Median age was significantly lower (p = 0.03) in patients receiving a tumor prosthesis (61.9 years, IQR 59.5 – 72.7) compared to the osteosynthesis group (73.8, IQR 66.5 - 80.5). Preoperative general state of health demonstrated by the Karnofsky performance status also differed significantly in favor of the prosthesis group (p = 0.01) and was judged moderate vs. poor, respectively. This was confirmed by evaluating preoperative ambulatory capacity. In the prosthesis group all patients were able to walk, whereas in the internal fixation group 58.3% of patients were wheel-chair or bed bound (Table 2). The median Tokuhashi score was 7.0 (IQR 6.3 – 8.0) and 6.0 (IQR 5.3 – 7.8) in the prosthesis and osteosynthesis group, respectively and did not differ significantly (p = 0.2). No intraoperative deaths occurred in either groups. Within the first 14 postoperative days three patients in the osteosynthesis group and one patient in the prosthesis group died. All preoperative ambulatory patients surviving the first two postoperative weeks resumed walking again (Table 2). Two patients in the intramedullary group complained about persistent pain. Both of them suffered implant failure three and five months postoperatively due to cut out and nail braekage. Conversion to a tumor prosthesis was accomplished in these two patients. The secondary implanted tumor prostheses required no surgical revision, although one patient developed a late deep infection with a fistula. This patient refused surgical intervention. At the last follow-up in 22 months after operation the stable fistula persisted with no additional systemic or local signs of infection. The patient is ambulatory and reports no pain, no signs of loosening were evident on x-rays. The other patient with an implant exchange died 28 months after revision. The third patient with a complication after osteosynthesis necessitated revision because of deep infection, accounting a for total of three complications (25%). In the prosthesis group two out of eight primary tumor prostheses (25%) needed conversion from hemi- to total hip arthroplasty or revision of the acetabular cup because of recurrent dislocation. Reconstruction of the capsule or use of an attachment tube was not performed in these two patients.

**Table 1:** Age, time periods and survival

	**Proximal femoral replacement**	**Osteosynthesis**	**p**
**Age at operation (years)**	61.9 (59.5 – 72.7)	73.8 (66.5 – 80.4)	< 0.05
**Time from initial diagnosis to operation (months)**	54.5 (4.8 – 87.3)	24.0 (1.0 – 64.3)	0.43
**Patient survival since operation (months)**	4.5 (2.3 – 16.5)	2.0 (0.3 – 20.5)	0.58
**Implant survival (months)**	2.5 (1.0 – 7.5)	3.0 (0.3 – 11.0)	0.93
**Implant durability (months)**	4.5 (2.3 – 16.5)	2.0 (0.3 – 11.0)	0.31
**Patient survival since initial diagnosis (months)**	65.0 (10.8 – 112.0)	30.0 (1.5 – 87.5)	0.34

Values are given as median values (interquartile range IQR). Implant survival reflects the time period from surgery until death, follow-up or revision for any reason. Implant durability refers to the time from surgery until death, follow-up or revision for structural implant failure only (i.e. nail breakage, periimplant/periprosthetic fracture, loosening, etc.).

**Table 2:** Preoperative health condition and ambulatory capacity

	**Proximal femoral replacement**		**Osteosynthesis**		**p**
**Karnofsky index**	65.0 (52.5 – 90.0)		45.0 (30.0 – 50.0)		< 0.05
**Takahashi score**	7.0 (6.3 – 8.0)		6.0 (5.3 – 7.8)		0.20
**Ambulatory capacity**	**preop.**	**postop.**	**preop.**	**postop.**	
**Normal (n)**	3	1	4	1	
**With walking aids (n)**	5	6	1	4	
**Wheel chair/bed bound (n)**	-		7	4	

Values for the Karnofsky index and Takahashi score are given as median values (interquartile range IQR). Ambulatory capacity is demonstrated by number of patients with different categories of mobility before and after surgery. Walking capacity was not evaluated in one patient after endoprothetic reconstruction and in three patients after intramedullary nailing, because of progressive disease and early postoperative death.

An additional analysis was performed accounting the two early osteosynthesis failures with implant exchange to the prosthesis group. Nevertheless, no significant differences with respect to patient survival and implant survivorship were found.

## Discussion

In terms of patient survival it is very difficult to estimate the role of the surgical method or choice of implant. Many cofactors have shown to influence patient survival in metastatic bone disease as age, preoperative general health status, type of cancer, location of metastasis in the femur or solitary versus multiple metastases [11-14]. Apart from these factors actual fracture compared to impending fracture in long bones seems to be another essential cause influencing patient survival [5,7]. Mavrogenis et al. [5] demonstrated that impending fractures show a significantly better life expectancy with survival rates of approximately 60% at one year, 40% at two years, 30% at three years and 20% after 5 years. Survival rates of actual pathologic femoral fracture are reported to be approximately 45% at six months, 30% at one year, 15% at two years and less than 10% after 3 years [4,5,15], corresponding well to our results. Mavrogenis et al. additionally found that the type of surgery is a significant factor in patient survival. This was also demonstrated for pathologic fractures of the proximal femur. Patient survival was significantly higher in 18 patients with proximal femoral resection and modular prosthetic replacement compared to 11 patients with intramedullary nailing [5]. According to these results our primary hypothesis was to confirm superior survival in patients undergoing proximal femoral resection and endoprothetic reconstruction compared to intramedullary osteosynthesis. Although we could see a tendency to better survival in the prosthetic group it was statistically not significant. Besides, preoperative general health performance which is a substantial predictor of survival in skeletal metastases [11,12] was significantly lower in the osteosynthesis group contributing to an earlier death and putting a better survival by trend in the prosthesis group into perspective. Preoperative general health performance was not reported by Mavrogenis et al. Consequently, the benefit in patient survival after resection and endoprosthesis in their study might be equivocal [5].

None of the patients in our series died because of intraoperative embolic events. It has been shown by others that implantation of a long intramedullary nail or a long-stem cemented femoral component in patients with femoral metastasis increases the risk of an embolic syndrome considerably resulting in catastrophic outcome in up to 8% [16-19]. We believe that cemented stems with a regular length and modular reconstruction bushings bridging the osseous defect as applied in our study may help in reducing the risk of an embolic syndrome und increase patient safety.

**Figure 3:**
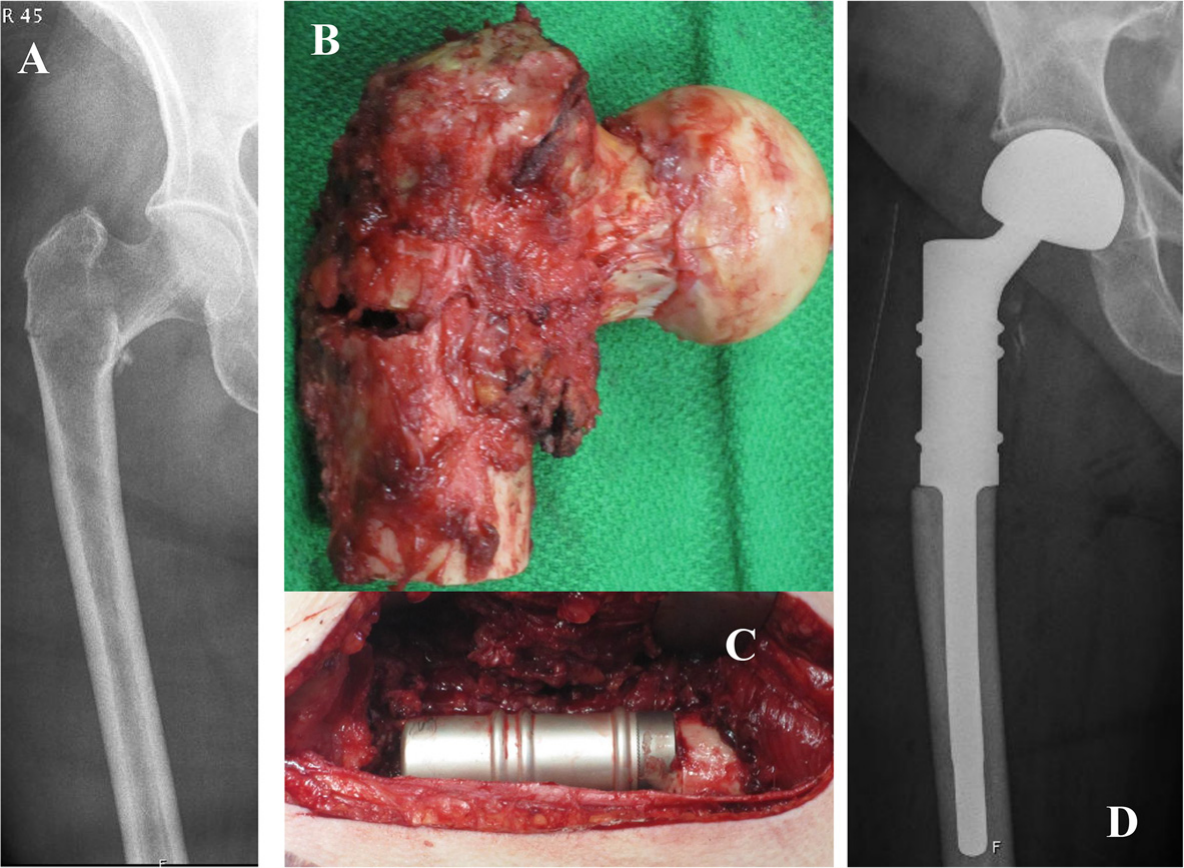
**Proximal femoral replacement arthroplasty. A)** Preoperative X-ray, a.p. view. **B)** Completely resected proximal femur including the pathologic fracture, soft-tissues and capsule left in situ. **C)** Intraoperative view of a proximal femoral replacement after reconstruction of the capsule. **D)** Postoperative X-ray showing the prosthesis with a bipolar head, a.p. view.

Complications requiring reoperation are reported to be as high as 26% for intramedullary nailing and 18% for endoprosthesic replacement in treatment of pathologic fracture of the proximal femur [4,9]. This compares well to our results with a complication rate of 25% in each group. As for intramedullary nailing complication rates might be substantially higher considering actual pathologic fractures exclusively. The rate of fracture union is considerably less than 50% for common tumor types such as breast and renal carcinoma or even absent as demonstrated for lung carcinoma [6]. On the other hand impending fractures proceed to fracture in only 13% after local irradiation [20], subsequently load induced stress to osteosynthetic devices and potential failure is conspicuously lower. This is confirmed by Harvey et al. [9] demonstrating a significantly higher complication rate for intramedullary nails in actual pathologic fractures than in impending fractures. Other authors report considerably lower complication rates for intramedullary nailing ranging from 2 to 6%, but it must be mentioned that impending fractures account for approximately 60-67% in these studies [5,7,8]. Many authors of larger series advocate arthroplasty in favor of osteosynthesis in metastases of the proximal femur because of superior durability and lower complication rates [4,8,9]. Our complication rate of 25% in the prosthesis group compared well to the results of Harvey et al. [9]. Nevertheless, this is higher than the 3-10% several others authors have reported [4,5,8]. A reason for our higher dislocation rate might be, that 50% received a total arthroplasty which is associated with substantially higher dislocation rates than hemiarthroplasty in this setting [4,21]. Most other studies use hemiarthroplasty in the vast majority of cases [4,8,9], explaining a lower complication rate in this respect. Additionally, regular or long-stemmed revision femoral components are widely used [4,8,22] preserving the greater trochanter and subsequently improving hip joint stability. In order to improve safety and reduce the higher dislocation risk of proximal femoral replacements, preservation and repair of the hip capsule as well as applying a bipolar head whenever possible is recommended [9]. If preservation of the capsule is not possible, attachment tubes for soft tissue reconstruction or tripolar cups might help in reducing the risk of dislocation [23]. Both options were not performed in our patients except in one (Figure 3).

Preoperative general health status is an important parameter in predicting survival in patients with skeletal metastases [11,12]. Unfortunately, many authors do not report on this [4,5,9]. As for our study groups we demonstrated a significant difference suggesting a profound selection bias. Correspondingly, most of the patients with a poor preoperative health condition were treated with an intramedullary nail and patients with moderate to good general performance received endoprosthetic reconstruction. It should be mentioned that two out of four patients with a good preoperative general health condition and normal ambulatory capacity in the osteosynthesis group sustained a hardware failure. This is in contrast to the results of Steensma et al. [8]. They reported a preoperative Eastern Cooperative Oncology Group (ECOG) Score of 2 points or less in 88% of patients treated with an intramedullary nail (IM) compared to only 61% in the endoprosthesis group. That means almost all patients in the IM group were ambulatory and had a moderate to very good preoperative health status preoperatively. Nevertheless, a good health status and a subsequently higher activity of patients did not contribute to a higher implant related complication rate after nailing which was 6.1% and is considerably lower than in our IM group. A possible explanation might be the relatively high rate of impending fractures (70%) as discussed above [8].

Despite the relatively small number of patients our study holds several strengths compared to other larger series [4,7-9]. First, we focused on the actual pathologic fracture and its corresponding characteristic features outlined above, exclusively. Second, we included only patients with multiple osseous metastases and fracture location in the proximal femur, excluding further potential confounding variables. Third, we addressed the preoperative general health performance which is a major prognostic factor in patient survival and correspondingly supported interpretation of survival data which is rarely reflected in other studies. On the other hand, several limitations of our study must be mentioned. First, the retrospective design and relatively small number of patients in the study comprise familiar limitations by itself. Nevertheless, actual pathologic fractures are quite rare and survival of patients is limited qualifying this type of study. Second, many different types of cancer were included. However, distribution of cancer type according to aggressiveness [12] was comparable with approximately 60% of slow to moderate growth types in each group. Third, adjuvant and neoadjuvant therapy was not considered. But a wide divergence of treatment protocols and unclear effectiveness [22] precluded inclusion of this criterion.

## Conclusion

In summary, pathologic fractures are treated equally safe by osteosynthesis using an intramedullary nail or proximal femoral resection and endoprosthetic reconstruction. Patient survival was not influenced by type of surgery or choice of implant. Patients that show a good to moderate general health status and are ambulatory preoperatively might benefit from primary endoprosthetic reconstruction due to longer implant durability. The only concerning complication after proximal femoral resection and endoprosthetic reconstruction in our series was dislocation which must be prevented in order to see a clear benefit over intramedullary nailing in patients with expected longer survival.

## Competing interests

All authors declare that they have no competing interests.

## Authors’ contributions

JKMF made substantial contributions for conception, design, analysis and interpretation of the data. FH did the main part in data acquisition. JB has been involved in drafting the manuscript. CJ has given final approval of the version to be published. All authors read and approved the final manuscript.
